# Contextual socioeconomic determinants of cardiovascular risk factors in rural south-west China: a multilevel analysis

**DOI:** 10.1186/1471-2458-7-72

**Published:** 2007-05-05

**Authors:** Cai Le, Virasakdi Chongsuvivatwong, Alan Geater

**Affiliations:** 1 191 Western Renmin Road, Department of Health Information and Economics, Faculty of Public Health, Kunming Medical College, Kunming 650031, China; 2 Epidemiology Unit, Faculty of Medicine, Prince of Songkla University, Hat Yai, Songkhla 90112, Thailand

## Abstract

**Background:**

We examined independent influences of contextual variables on cardiovascular risk factors in Shilin county, Yunnan province, South-west China.

**Methods:**

Three villages were selected from each of the ten townships based on probability proportional to size. In each selected village, 200 individuals aged ≥ 45 years were chosen based on simple random sampling method. From 6006 individuals, information on demographic characteristics, smoking and drinking status was obtained by interview. Blood pressure, height, weight, and waist and hip girth were measured. Fasting blood sugar was measured in a 10-percent subsample. Contextual data were from official reports. Multi-level regression modelling with adjustment for individual and contextual variables was used.

**Results:**

Contextual variables associated with CVD risk factors included: remoteness of village with higher blood pressure and fasting blood sugar, high proportion of Yi minority with drinking, high literacy rate with a lower rate of smoking and a lower mean waist-hip ratio, and high average income with lower systolic blood pressure and body mass index (BMI) but higher FBS.

**Conclusion:**

While contextual SES is associated with a few CVD risk factors, villages with high level of income are worse off in fasting blood sugar. Strategies of economic development should be reviewed to avoid adverse effects on health.

## Background

Cardiovascular diseases (CVD) are a major public health concern in the world, accounting for half of all non-communicable disease deaths worldwide [1]. Similar to findings from western countries [2,3], risk factors for CVD in many developing countries have been well recognized [4,5]. Diabetes, hypertension, cigarette smoking, alcohol drinking and overweight have been found to be major risk factors for CVD in China [6-8]. There is also growing evidence that the prevalence of CVD risk factors has been increasing, and clustering of CVD risk factors is common in China [9,10].

Traditionally, epidemiological studies have focused on identifying individual-level risk factors for diseases. Recently in epidemiology, there is increasing interest in exploring the effect of population or group (contextual) variables on disease risks. People residing in the same community or context would share the same contextual or environmental exposure and statistical assumption of independence is usually not true. Without proper adjustment, simplistic analysis of contextual independent variables as if they vary independently across individual subjects would bias the result toward overestimation of association. Multilevel modelling [11] provides a useful solution to simultaneously examine the effects of individual-level and contextual-level variables.

Discrimination of effects of contextual from individual independent variables is also important for public health. A good example of contextual concept is externality. While individual socioeconomic status (SES) variables such as income, unemployment and educational level [12-16] have been recognized to be important determinants of CVD risk factors, little is known about the influence of socioeconomic status of the neighbourhood. If such effects exist and are stronger than individual SES, all the residents would have a better health if they cooperate to raise the community SES than just compete to get to the most without making a contribution to local society. Another example would be independent effects of individual versus contextual ethnicity. CVD risk factors are associated with ethnicity [17] either through culture or genetics or both. As culture is contextual, one would expect strong effect of contextual ethnic effect if it is the main mechanisms. On the other hand, independent influence of genetic can be expressed as an individual ethnic effect. This is independent from the ethnic of the community where the subject resides.

Yunnan province of China is a multi-ethnic area and has 52 ethnic groups. The terrain is mainly mountainous with high variability in level of socioeconomic development and ratio of ethnicities among communities. The risk for CVD among the population is high and also varies by geography [18]. Under such circumstance, our purpose in this study was to test the independent effects of contextual socioeconomic variables with adjustment for individual socioeconomic variables on risk factors for CVD in this study area. Understanding characteristics of village associated with CVD risk factors can assist health planning to allocate appropriate resources to the target area.

## Methods

### Study design

This was a cross-sectional community survey combined with investigation of contextual variables from existing official data sources.

### Study area and population

Shi Lin County, a rural area of Kunming, the capital of Yunnan province (one of the poorest provinces in south-west China), was chosen as the study community. In 2004, it had a population of 205,186 and contained 10 townships, 90 villages and 65,135 families. The county was a typical minority rural county in Kunming, predominated by Yi ethnic group, and is one of the poor counties in Kunming, with a per capita income among peasants of US$313 [19] in 2004. The total area was 1717 square kilometers, and mostly mountainous. Villages were scattered with a maximum distance of 80 kilometers from the main city, Kunming. Transportation by car was available throughout the year.

### Data source

Socioeconomic characteristics of villages with regard to population size, adult literacy rate, proportion of Yi ethnic minority and distance from city were based on the 2000 census in China obtained from the local statistics office. Individual characteristics, village average income and CVD risk factor parameters were obtained from a cross-sectional community survey.

### Community survey

#### Sampling technique

In order to obtain information on each township for subsequent spatial study, reported separately, standard cluster sampling technique [20] was modified to select three clusters or villages from each township. Altogether there were 30 study villages.

In each selected village, from a name list of individuals aged ≥ 45 years obtained from the village committee, 200 subjects were chosen based on simple random sampling method.

### Data Collection and measurement

The questionnaire was adapted from that used by Inter-ASIA collaborative group [4]. The instrument was modified to suit the local situation. Twenty fifth-grade medical students from Kunming Medical College were trained to be data collectors under the supervision of two senior medical staff. The training included introduction to cardiovascular diseases, use of the questionnaire, conducting fasting blood sugar test and taking anthropometric measurements.

In the morning of data collection, each participant was given full explanation of the research purpose, invited to participate and sign informed consent, and interviewed by one of the interviewers. Information on demographic characteristics, status of current cigarette smoking and alcohol drinking, blood pressure and fasting blood sugar test was obtained. Anthropometric measurements included height, weight, and waist and hip girth. Data collection was started in May 2005 and completed within 45 days.

Three blood pressure (BP) measurements were made according to the American Heart Association recommendations [21]. After at least 5 minutes of rest in a sitting position, systolic and diastolic pressures were taken from the participant's right arm, using a mercury sphygmomanometer. In our study, BP measures were based on the average of three BP readings. Our method is slightly different from Health Survey for England/Scotland standard and MONICA standards [22], which keep the average of the second and third measurements and reject the first.

Weight was measured using a balance beam scale. The measurement of height and weight was done with the participants standing on the scale wearing indoor clothes and barefoot. To ensure sufficient precision, height was measured to the nearest 0.2 centimeter, and weight was measured to the nearest 0.2 kilogram. Body mass index (BMI) was calculated as weight in kg divided by height in meters squared [23].

Waist girth was measured around the narrowest point between ribs and hips when viewed from the front after exhaling. Hip girth was measured at the point where the buttocks extended the maximum when viewed from the side. Waist-hip ratio was subsequently calculated.

A random 10% of participants were selected from each village for fasting blood sugar study. The subjects were instructed to fast overnight for at least 10 hours. A small drop of fingerprick blood was obtained and put onto a special strip of paper, which was then inserted into the blood glucose monitor machine (Braun, Beijing NecKar Healthcare Company). The monitor measured and displayed the result within 15 seconds. This strip glucose technique had been validated against a standard test in Kunming Hospital, and found to give a 10% lower value. Values obtained from the participants' blood stick test were therefore adjusted upward as appropriate.

### Ethical approval

This study was approved by the Ethics Committee of Faculty of Medicine, Prince of Songkla University, before carrying out the research.

### Definitions

Cigarette smokers were defined as persons who had smoked at least 100 cigarettes in their lifetime, and those who smoked tobacco products during the survey time were classified as current smokers. Current drinkers were defined as persons who drank alcohol regularly during the previous 12 months (i.e. on 12 or more different days during the year). Hypertension was defined as a mean systolic blood pressure ≥ 140 mm Hg, diastolic blood pressure ≥ 90 mm Hg, and/or use of antihypertensive medications. Overweight was defined as a BMI of 25 kg/m^2 ^or greater [23]. Diabetes mellitus was defined as a fasting plasma glucose ≥ 7.0 mmol/l (126 mg/dl) [24] or the use of antidiabetic medications. Adult literacy rate was defined as the percentage of population aged 15 years and over who could both read and write with understanding a short simple statement on his/her everyday life.

### Outcome variables

The outcome variables included systolic blood pressure (mmHg), diastolic blood pressure (mmHg), fasting blood sugar (mmol/l), BMI (kg/m^2^), waist-hip ratio, current smoker and current drinker.

### Independent variables

Individual-level independent variables were age, sex, ethnicity, household income and education, which are known socio-economic risk factors. The contextual variables included population size, adult literacy rate, proportion of Yi ethnic minority, mean income and distance from city.

### Statistical analysis

Mean income of participants from each village was computed for using as a contextual variable since this information was not available in any report. Descriptive statistics were used for data summary. Age-sex-adjusted prevalence of each CVD risk factor was computed by indirectly standardizing to the overall sample [25]. The data were further analyzed using multilevel regression. The method of estimation was by constructing a Generalized Linear Model using Penalized Quasi-Likelihood, with individual characteristics at the first level and village socioeconomic status at the second. We examined all individual variables and fitted models for each of the village variables separately. Both individual and village characteristics were treated as fixed effects. Multilevel logistic regression was used to analyze the association between independent variables and binary individual outcome such as current smokers and drinkers, whereas multilevel linear regression was used to analyze CVD risk factors which had a continuous distribution such as blood pressure and fasting blood sugar. The levels of association were expressed as standardized beta coefficients and standard errors to allow easy comparison across different independent variables. All data analyses were done with R software, version 2.1.1 [26].

## Results

A total of 6050 individuals aged ≥ 45 years was selected by the sampling process. Of these, 6006 participated (response rate = 99.3%). Among the participants, 611 randomly selected people received fasting blood sugar test. Their demographic characteristics are summarized in Table 1.

**Table 1:** Demographic characteristic of the study participants

Variables	n	%
Sex		
Male	2905	48.4
Female	3101	51.6
Age		
40–49 years	1102	18.3
50–54 years	1106	18.4
55–59 years	879	14.6
60–64 years	909	15.1
≥ 65 years	2010	33.5
Ethnicity		
Han	3664	61.0
Yi ethnic minority	2272	39.0
Educational level		
Illiterate	3328	55.4
Primary (grade 1–6) or higher	2678	45.6
Approximate yearly household income (Yuan)		
Max	20000	
P_75_	7000	
P_50_	4000	
P_25_	2000	
Min	1100	

Individual mean values of height, weight, waist girth, hip girth, BMI, waist-hip ratio, blood pressure and fasting blood sugar by sex are demonstrated in Table 2. In general, men had slightly higher mean values of BMI, waist-hip ratio, systolic pressure (SBP), diastolic pressure (DBP) and fasting blood sugar than women.

**Table 2:** CVD risk factor parameters by sex (mean ± SD*)

Variables	Male	Female	All
Height (cm)	162.3 ± 7.4	153.7 ± 6.6	157.9 ± 8.2
Weight (kg)	58.7 ± 8.8	50.6 ± 7.8	54.5 ± 9.2
BMI** (kg/m^2^)	22.3 ± 3.1	21.4 ± 3.0	21.8 ± 3.0
Waist girth (cm)	76.7 ± 8.2	66.8 ± 8.5	71.6 ± 9.7
Hip girth (cm)	87.0 ± 7.8	85.5 ± 7.9	86.2 ± 7.9
Waist-hip ratio	0.88 ± 0.07	0.78 ± 0.06	0.83± 0.08
Systolic pressure (mmHg)	120 ± 16	117 ± 17	118 ± 16
Diastolic pressure (mmHg)	79 ± 11	76 ± 11	77 ± 11
Fasting blood sugar (mmol/l)	5.5 ± 1.1	5.4 ± 1.0	5.4 ± 1.1

* Standard deviation

** Body mass index, which has been promulgated by the World Health Organization as the most useful epidemiological measure of obesity.

Table 3 summarizes the village contextual variables. Variations in adult literacy rate and percentage of minority ethnic were high.

**Table 3:** Distribution of socioeconomic status among 30 villages

Variables	Max	P_75_	P_50_	P_25_	Min
Population size	7217	3343	2410	1790	1120
Adult literacy rate* (%)	73.0	54.5	47.0	32.5	29.0
Proportion of Yi ethnic minority (%)	97.0	64.8	33.5	4.8	1.5
Average yearly income (Yuan)	9200	7500	6200	5725	4900
Distance from city (km)	49.0	33.2	16.3	8.0	0.9

* The percentage of population aged 15 years and over who could both read and write with understanding a short simple statement on his/her everyday life.

Table 4 shows the distribution of age-sex-adjusted prevalence of CVD risk factors among the 30 villages. Males had somewhat higher prevalences of hypertension, diabetes and overweight and remarkably higher prevalences of current smokers and current drinkers than females.

**Table 4:** Distribution of age – and sex-adjusted prevalence (%) of CVD risk factors among 30 villages

Variables	Male					Female				
		
	Max	P_75_	P_50_	P_25_	Min	Max	P_75_	P_50_	P_25_	Min
Hypertension	33.8	27.3	20.5	12.8	10.3	32.4	25.3	17.0	11.2	10.2
Diabetes	9.8	9.1	7.7	4.4	4.0	9.1	8.3	5.7	4.8	3.8
Overweight	24.5	21.4	14.7	10.0	9.5	20.6	15.8	11.8	9.7	8.9
Current smokers	72.0	65.3	52.0	33.5	31.0	3.0	2.0	1.0	0.2	0.2
Current drinkers	68.3	58.2	50.0	41.1	35.0	6.7	3.6	1.4	0.3	0.2

From results of multi-level analysis are shown in Table 5. Residents of villages with large population size had increased mean individual SBP and waist-hip ratio. Those in communities with low literacy rate had increased individual waist-hip ratio and probability of smoking habit. Living in a village dominated by Yi ethnic minority increased individual probability to be a drinker. Subject in low income villages had increased SBP and BMI, but had decreased fasting blood sugar. Individuals in remote villages had increased mean SBP, higher fasting blood sugar and increased probability of being a current smoker.

**Table 5:** Contextual effect of village characteristics on the CVD risk factors after adjustment for individual characteristics

Predictors	Systolic pressure (mmHg)	Diastolic pressure (mmHg)	Fasting blood sugar (mmol/l)	BMI	Waist-hip ratio	Current smokers^†^	Current Drinkers^††^
							
	Standardized Beta^††† ^(SE^‡^)	Standardized Beta (SE)	Standardized Beta (SE)	Standardized Beta (SE)	Standardized Beta (SE)	Standardized Beta (SE)	Standardized Beta (SE)
**Contextual variables:**							
Population size (X1000)	0.08* (0.04)	0.02 (0.07)	-0.08 (0.07)	0.09 (0.05)	0.10* (0.05)	0.08 (0.28)	0.14 (0.10)
Adult literacy rate (%)	0.006 (0.04)	0.01 (0.05)	0.11 (0.06)	0.02 (0.04)	-0.12**(0.04)	-0.57* (0.24)	-0.03 (0.09)
Proportion of Yi ethnic minority (%)	-0.09 (0.05)	-0.12 (0.08)	0.08 (0.08)	-0.03 (0.06)	-0.09 (0.05)	0.50 (0.33)	0.43*** (0.12)
Income (× 1000 Yuan)	-0.09* (0.04)	-0.07 (0.07)	0.34*** (0.07)	-0.09* (0.04)	0.02 (0.04)	0.57 (0.29)	0.11 (0.10)
Distance from city (km)	0.11*(0.04)	0.04 (0.07)	0.16* (0.07)	0.03 (0.05)	-0.02 (0.04)	0.75** (0.30)	0.08 (0.11)
**Individual variables:**							
Age	0.11*** (0.01)	0.06** (0.01)	0.07* (0.03)	-0.09**(0.01)	0.02* (0.01)	-0.12** (0.04)	-0.17***(0.03)
Sex (reference: male)	-0.20*** (0.03)	-0.19** (0.03)	-0.02 (0.08)	-0.28**(0.03)	-1.24** (0.02)	-4.91***(0.23)	-3.86***(0.14)
Educational level (reference: illiterate)	-0.02 (0.02)	-0.02 (0.02)	0.09 (0.06)	-0.003 (0.02)	0.01 (0.02)	0.06 (0.07)	0.04 (0.05)
Ethnic (reference: Han)	0.11** (0.03)	0.01 (0.04)	0.21 (0.11)	-0.13** (0.04)	0.04 (0.03)	0.04 (0.14)	0.29** (0.11)
Income (× 1000 Yuan)	-0.01** (0.002)	-0.007** (0.002)	0.001 (0.001)	-0.01**(0.002)	0.002 (0.002)	0.007 (0.007)	0.01 (0.008)

* p < 0.05, ** p < 0.01, *** p < 0.001, † reference: non-current smokers, † † reference: non-current drinkers, † † † regression coefficient. ‡ standard error.

## Discussion

From this study, several geographical and socio-economic contextual variables were shown to exert an independent influence on CVD risk factors.

While a previous study found high prevalence of CVD risk factors in poor rural villagers who were rarely exposed to the modern world [27], our findings that distance from the city is positively associated with SBP, fasting blood sugar and smoking do emphasize the need for more attention to the CVD problem in remote areas. Remoteness of the village in the study area is somewhat correlated with high altitude, which has been shown to cause an increase in blood pressure (BP) in other studies [28,29]. A study from Taiwan indicated that people living in mountainous areas have the highest prevalence of diabetes compared to those living in metropolitan cities [30]. The mechanism of association therefore could be through physiological and/or lifestyle aetiology, and needs further investigation.

While an inverse relationship between individual educational level and CVD risk factors has been reported in a number of studies [31-33], only a few have explored the effect of education at the contextual level and reported associations with various CVD risk factors, most of them in the direction not favourable for those living in poor education areas, such as an increased risk for smoking and increases in SBP [34] and diastolic pressure [35]. Our study yielded no evidence supporting any association between low individual educational level and CVD risk factors but a strong preventive effect on smoking and waist-hip ratio of increased literacy rate. In these rural communities, collective social life style is very strong. Influence of peers and neighbourhood on smoking and social activity, such as poor diet and lack of exercise, leading to obesity may overwhelm the effect of individual education. The inverse association of literacy rate with these two factors suggests that the educated communities rather than the uneducated ones have until now been the target for intervention programmes.

Yi ethnic minority has increased risk for alcohol drinking in this study. Alcohol drinking is an integral part of Chinese culture. Distilled spirit is the primary beverage of choice for men, accounting for more than one-third of all drinks consumed [36]. A survey conducted in three centers of China indicated reasons for drinking were alcohol's positive social effects, relief of tension and worry, and relief of craving and withdrawal symptoms [37]. The phenomenon that ethnic minorities have more frequent reported alcohol intake than Han majority has been demonstrated in other studies [38,39]. In our study, while individual Yi ethnicity has a positive relationship with alcohol drinking and blood pressure and negative relationship with BMI, community ethnicity has an effect only on drinking and not on other risk factor. These results thus link culture only with alcohol and not with other CVD risk factors.

While western studies have demonstrated that deprivation in low income communities increases the level of various CVD risk factors such as SBP [34], overweight [40] and diabetes [41], our results agree with them on the protective effect of income against SBP and BMI but by contrast demonstrated an adverse effect on fasting blood sugar. This adversity is from village income and not individual income. This suggests that contextual lifestyle of the relatively rich communities must be investigated and appropriate intervention applied. Public environmental factors demanding more physical exercise such as transportation by bicycles and walking and predominant manual occupation such as farming may be more common in low income communities.

The strength of our study lies in the large sample size and high response rate fulfilling the requirement for multilevel modelling [9]. The limitation of this study is that fasting blood sugar test was not done for all participants and none of the lipid profiles was available due to financial difficulties. Capillary blood glucose has slightly lower reading than plasma glucose level obtained from venous blood. Nevertheless, the relationship of blood sugar with contextual SES is illustrated, although further studies are needed to confirm these findings. Furthermore, social equity index such as Gini's coefficient for each community was not available. Thus we were unable to test the effect of local social equality on CVD risk factors.

## Conclusion

The study area shares the global trend of increased CVD risk factors, which are contributed to by contextual setting in addition to the known personal lifestyles. Unlike findings from the western countries, where most unfavourable outcome are more common among the low SES, local directions of association are inconsistent. While contextual SES development is associated with a few CVD risk factors, villages with high level of income are worse off in fasting blood sugar. Strategies of economic development should be reviewed to avoid their adverse effects on health.

## Competing interests

The authors declare that they have no competing interests.

## Authors' contributions

Cai Le carried out the study and drafted and revised the manuscript. Virasakdi Chongsuvivatwong conceptualized the research idea, participated in the design of the study, laid out the framework for data analysis, interpreted the results and helped to draft the manuscript and revised the discussion. Alan Geater participated in the design of the study and helped to draft and revised the manuscript. All authors read and approved the final manuscript.

## Pre-publication history

The pre-publication history for this paper can be accessed here:


